# Graduate students locked down? PhD students’ satisfaction with supervision during the first and second COVID-19 lockdown in Belgium

**DOI:** 10.1371/journal.pone.0268923

**Published:** 2022-05-23

**Authors:** Theun Pieter van Tienoven, Anaïs Glorieux, Joeri Minnen, Petrus te Braak, Bram Spruyt

**Affiliations:** Research Group TOR, Sociology Department, Vrije Universiteit Brussel, Brussels, Belgium; Universidad Nacional Autonoma de Nicaragua Leon, NICARAGUA

## Abstract

**Background:**

Supervisor support is crucial for the successful and timely completion of the PhD and the largest contributor to PhD students’ overall job satisfaction. The COVID-19 pandemic affected PhD students’ life substantially through delayed experiments, missed timelines, running out of funding, change to online team- and supervisor meetings, mandatory working from home, and social confinement.

**Aim:**

This contribution considers PhD students’ satisfaction scores to reflect the extent to which PhD students felt supported by their supervisor during the COVID-19 pandemic so far and aims to investigate to what extent did PhD students’ satisfaction with supervisor support changed over time.

**Method:**

It uses two longitudinal two cohorts of wave 4 to 5 of the PhD Survey at a Belgian university. These cohorts are representative of two different ways the COVID-19 pandemic might have impacted doctoral research. Cohort 1 (n = 345) includes a pre-COVID measurement (April-May 2019) and a measurement immediately after the start of the abrupt lockdown in April-May 2020. Cohort 2 (n = 349) includes the measurement at the onset of the pandemic in 2020 and after a year with continuously changing containment policies (April-May 2021). The composite measure of satisfaction with supervisor support is based on six items with high internal consistency.

**Results:**

No significant net effect of time was revealed. Instead within subject interactions with time showed that in cohort 1, PhD students at the start of their PhD trajectory and PhD students with family responsibilities reported lower supervisor satisfaction scores over time. In cohort 2, PhD students not pursuing academic careers reported lower satisfaction scores over time.

**Conclusion:**

In times of crises, special attention needs to be paid to PhD students who are extra susceptible to uncertainties because of their junior status or personal situation, and especially those PhD students for whom doctoral research is not a trajectory to position themselves in academia.

## Introduction

At the time the SARS-CoV-19 virus took hold of the world (early 2020), it was not clear how the COVID-19 pandemic would unfold over the years. At the onset there was an abrupt, chaotic, and very strict lockdown. Gradually, fluctuations in infections led to a varying policy of tightening and easing. Like so many others, PhD students are also confronted with the impact of the COVID-19 pandemic. This impact translates into uncertainties about being able to carry out research in the set research period, into the loss and/or digitization of the intellectual and social support of colleagues and supervisors, and into challenges of combining PhD research with impacted responsibilities in family and personal life.

Concerns about the impact of the COVID-19 pandemic are mainly vocalized in (bio)medical and sciences disciplines [1–5]. On the one hand, these disciplines rely heavily on experiments in laboratories that are not so easily postponed or on face-to-face medical investigations that could not proceed because of social distancing rules. On the other hand, these disciplines tend to have the academic tradition to write commentaries and letters to editors. Undoubtedly, PhD students in *all* academic disciplines are in precarious statutes and are faced with the uncertainties and difficulties of the COVID-19 pandemic. Similar, *all* academic disciplines will have reasons to expect a negative impact on their PhD research. Therefore, a comparison of disciplines and the different type of research that characterizes these disciplines, is recommended.

Regardless of the disciplines, supervisor support is crucial for successful completion of a PhD [6] and expected to be even more important in the unprecedented research environment created by the COVID-19 pandemic [7, 8]. Especially because supervisor support can take away uncertainties and positively contribute to PhD students’ well-being [9, 10]. For these reasons, the evaluation of supervisor support by PhD students can be considered an important indicator of how supervisors’ efforts and abilities mitigate the impact of the COVID-19 pandemic.

Against that background, this contribution aims to answer the following question: to what extent did the COVID-19 pandemic impact PhD students’ satisfaction with supervisor support during the abrupt onset and during a year of alternating tightening and easing of restrictions? We will use longitudinal data of two cohorts. Cohort 1 (April-May 2019 –April-May 2020) represents a pre-COVID measurement and a measurement immediately after the start of the abrupt lockdown (March-May 2020). Cohort 2 (April-May 2020 –April-May 2021) represents a measurement at the onset of the pandemic and after a year with continuously changing containment policies. Moreover, our data are conducted university wide, which allows to compare all academic disciplines.

## Background

### Supervision and satisfaction among PhD students

PhD students are assessed on their thesis, whether after an oral defence, and this process typically takes four years [11]. During this process, the support of supervisors in terms of expertise, time, and support is essential [12]. The pedagogical aspect of supervision plays a key role in the successful and timely completion of the PhD trajectory and is considered to be closely related to supervisors’ ideas of the purpose of doing doctoral research [11, 13]. Training PhD students to become independent and innovative researchers happens through learning research skills, requirements, and the ability to create new ideas, whereas enabling PhD students to develop as individuals happens through motivating PhD students in frequent meetings and taking feedback on supervisory arrangements [13].

Supervisory arrangements are said to “make or break” PhD students [6]. Not surprisingly, then, supervisors are the largest contributor to PhD students’ overall job satisfaction [9]. The expertise and scholarly ability, as well as the more personal supporting role of the supervisor play an important role here [14, 15]. The frequency and quality of the meetings, the encouragement, support, and feedback to publish, and opportunities to attend research seminars all contribute to the satisfaction of PhD students [12, 15]. However, ultimately, supervisors’ supportiveness trumps supervisors’ academic qualities as the main contributor to PhD students’ satisfaction [9].

It is not entirely inconceivable that a higher degree of satisfaction with the support of their supervisor also leads to a higher degree of well-being among PhD students [9]. All the more so because a large-scale, international study shows that large numbers of PhD students who had experienced depression and/or anxiety disagreed with statements about sufficient support from their supervisors [10]. Moreover, disagreement about or disruptions in supervisory arrangements cannot be ruled out [16]. In fact, common disagreements relate to supervisors not being involved in research decisions and PhD students judging supervisors not being up-to-date and providing dubious advice [17]. Disruptions in the relationship between supervisors and PhD students often relate to the duality in supervision situation because the tutoring relation and the supportive, more personal relation may interact negatively [18]. In addition, the COVID-19 pandemic forced universities to accelerate the implementation of a digital learning environment which implied additional challenges for establishing and maintaining dependency relations [19]. Digital competences [20], the quality of the digital leaning environment, and the engagement to this environment [21] will add to the complexity of supervisor arrangements. A ‘match’ between PhD students and supervisors in both the personal and academic relationship is crucial for completion rates and increased PhD students’ satisfaction [22].

### The impact of the COVID-19 pandemic

The precariousness of this balance between expected and obtained support from supervisors, between PhD students’ and supervisors’ professional and personal relationships, and the substantial impact this has on the successful completion of PhD research on the one hand and the well-being of PhD students on the other, was further emphasized at the onset of the COVID-19 pandemic. Numerous commentaries and editorial articles exposed the difficulties that PhD students encountered due to the pandemic: delayed experiments, missed timelines, running out of funding, change to online team- and supervisor meetings, mandatory working from home, social confinement (especially for foreign researchers), and the need for a supportive, divers and inclusive research community [1–5]. Following this call for attention to the well-being of scientists and PhD students during the COVID-19 pandemic, several studies followed that began to explore these challenges and threats further [7, 8, 23–28]. PhD students’ worries and concerns tend to fall into three categories [8].

#### Disruption of and changes in research activities

The COVID-19 pandemic forces some PhD students to alter their research designs and exposes them to the risk of going in overtime in times when funding becomes more scarce [8]. Moreover, the COVID-19 pandemic affects research designs unequally. PhD students that planned electronic data collection or had already collected data might even benefit from the COVID-19 pandemic because it causes people to familiarize with online technology or because it left PhD students with more time to spend on writing their theses [23]. Regarding the latter element Paula [4], however, warns that mandatory working from home in a crisis situation that extends beyond the realm of work cannot be equated with a boost in productivity. Indeed PhD students report a decrease in productivity [8] and an increase in workload [23].

#### Personal concerns

PhD students report not only being worried about the immediate as well as the long-term impact of the COVID-19 pandemic on their own health, but also on the health of friends and family [8]. In addition to health concerns, PhD students also report financial concerns. Indeed, research in Australia, for example, finds that the COVID-19 pandemic exacerbates the financial precarity of PhD students, with many considering quitting their research [26]. Finally, there are concerns about maintaining social connectedness [8]. Face-to-face networking with peers facilitates support such as problem solving and personal development [29]. Additionally, PhD students report meeting with friends and family as a coping strategy for stress [30].

#### Career impact

Research on the career impact of the COVID-19 pandemic shows ambiguous results. Quantitative data from 2^nd^ and 5^th^ year PhD students, for example, report that the COVID-19 pandemic, which thoroughly shook up the academic job-market, hardly changes the career aspirations of PhD students [25]. Contrarily, qualitative data from master students, PhD students, and postdoctoral researchers, report that PhD students are concerned about their competitiveness as a researcher and consider not pursuing a career in academia [8]. This ambiguity might result from different samples and methodology, but it might also relate to PhD students’ motivation to conduct PhD research. PhD students motivated by a professional quest (i.e., to derive professional advantages in terms of employment prospects or working conditions) or PhD students motivated by a fundamental desire for self-actualization are less likely to have academic career aspirations compared to PhD students motivated by an intellectual quest [31]. Additionally, this impact might be mediated by the academic discipline since, for example, medical PhD students tend to be much more motivated by career building aspirations than PhD students in sciences [32].

The impact of the preceding elements might be twofold. On the one hand, these worries and concerns contribute to PhD students’ stress levels, which are usually already high [30]. On the other hand, PhD students’ might expect additional support from their supervisors as a result from the COVID-19 pandemic. This expected additional support can be grouped in two categories [8]: understanding and empathy on the one hand and guidance and direction on the other hand. The former deals with understanding for delays, decreased productivity, and moral support to get back on track. The latter deals with the structural support such as adjusting their research plan, flexibility in timelines, and financial support.

Although the status of PhD students is sometimes considered precarious due to financial and time constraints, research reports a chasm in support for PhD students in relation to the COVID-19 pandemic [7]. One group reports unchanged support–a few even report improved support –and the other group reports worsened support. Surprisingly, these groups do not differ significantly by gender, living situation or year of their PhD trajectory. However, the group that reports worsened support is characterized by significantly larger shares of PhD students that saw a decrease in the frequency of supervision, that did not meet with their supervisor in person, that also witnessed a decrease in supervision via email messages, and that did not receive help from their supervisor to cope with COVID-related restrictions during the pandemic [7].

### The case of PhD students in Belgium

This study contributes to the growing body of knowledge on the impact of the COVID-19 pandemic on well-being of PhD students in a unique way. We will analyse PhD students’ satisfaction with supervisor support in two cohorts that are representative of two different ways the COVID-19 pandemic might have impacted doctoral research.

The first cohort concerns PhD students that were conducting doctoral research in April-May 2019 and in April-May 2020. This cohort was confronted very abruptly with a strict lockdown imposed by the Belgian Federal Government, and which reached its height on 18 March 2020 with the complete closure of schools and borders for non-essential travel. Social contacts had to be limited as much as possible and ‘contact bubbles’ were imposed. In no time universities switched to ‘code red’ which lasted until the end of the academic year (mid-July 2020). For many PhD students code red meant mandatory working from home, closure of all on campus facilities, and online contacts with supervisors and colleagues. Additionally, data collections had to be interrupted, postponed, or redesigned, because face-to-face interactions were not possible and laboratory use was scaled down to take into account social distancing regulations. Conferences, workshops, and other courses were cancelled or entirely took place online.

However, although the first lockdown was extremely disruptive for work and family life, with the summer of 2020 and the development of vaccines on the horizon, hope arose that this lockdown was a one-off. From July 2020 onwards, almost all restrictions were eased and the academic year of 2020–2021 started with hope.

This turned out to be a vain hope. From October 2020 onwards, the number of cases that tested positive for the SARS-CoV-19 virus started to rise again. The school autumn break was extended until mid-November and new restrictions were put in place. The second cohort represents PhD students that were conducting doctoral research in April-May 2020 and in April-May 2021. After facing a sudden lockdown, this cohort is characterized by an academic year that alternated between ‘code orange’ and ‘code red’ with varying restrictions on the number of days allowed to return to the workplace, the number of colleagues allowed to meet in person or to operate in laboratories, the possibilities to provide onsite, hybrid, or online teaching, and the partial opening of campus life. In other words, the shock effect of the first lockdown turned into a yearlong period of uncertainty, unpredictability, and great stress on the mental resilience of PhD students.

### Expectations

Based on the existing literature and the particularities concerning the way the pandemic evolved, we hypothesize that the first lockdown in 2020 has a substantial negative effect on PhD students’ satisfaction with supervisor support when compared to 2019 (**H1a)**. The lockdown of 2020 was unprecedented and both PhD students and supervisors not only had to cope with changes in the modus operandi of supervision and research plans, but also with the challenges of personal situations. We consider the difference between satisfaction scores of 2019 and 2020 to reflect the extent to which PhD students felt supported during these abrupt events. We hypothesize that the second lockdown of 2021 has a less substantial negative effect on PhD students’ satisfaction with supervisor support when compared to 2020 (**H1b**). Although it remained a year of relaxation and tightening of measures to combat the COVID-19 pandemic, the chaotic nature of the first lockdown will have partly given way to acquiesce in the situation, however disruptive it still has been. As such, we consider the satisfaction scores of 2021 to reflect the extent to which PhD students felt supported during the academic year full of uncertainties.

In addition to the hypothesized shift in PhD students’ satisfaction scores, we expect certain characteristics to have an additional direct or indirect influence on conducting doctoral research and which can therefore be a reason to expect (even) more support from supervisors.

#### Discipline

The type of doctoral research and especially, the collection of research data, may vary across disciplines, which, in turn, may have been impacted differently by the consequences of the COVID-19 pandemic. It is plausible that PhD students expect (extra) support from supervisors in solving these research related problems and in adapting their research plan and are therefore stricter in their assessment of the support they received from their supervisor. However, we expect little variation between the faculties, precisely because each department has its own problems when doing doctoral research during the COVID-19 pandemic (**H2**). Additionally, pre-COVID-19 research shows that different elements of supervisory might cancel each other out in an overall satisfaction score. Indeed, PhD students in humanities and social sciences tend to put more value on academic advising and a personal touch, whereas PhD students in biological and physical sciences strongly assess not being used as cheap labour and, together with their peers in social sciences, put more value on career development [15].

#### Phase of doctoral research

PhD students that are in the finalizing phase of their doctoral research are much more likely to know the ins and outs of academia than PhD students that just started. The latter might not only need more intellectual support to get their research started, but also more administrative support to find their way. In the absence of colleagues due to mandatory working from home or alternating days at work, these PhD students might expect (extra) support from supervisors. Similarly, PhD students that are in the executing phase of their doctoral research (i.e., collecting data) face several uncertainties and thus, might also expect (extra) support from supervisors. Although one study does not report differences based on year of research [7], another study makes notion that PhD students in their post-data collection phase might have less concerns [23]. Therefore, we hypothesize that the expectancy of (extra) support results in a stricter assessment of satisfaction with this support. In other words, we expect the satisfaction scores of PhD students that are not in the finalizing phase not only to be lower than their peers who are in the finalizing phase of their PhD (**H3a**) but also to decrease more over time (**H3b**).

#### Career aspirations and motivations

Motivations to embark on a PhD trajectory vary and relate to different career aspirations [31], which in turn may be impacted differently by the COVID-19 pandemic [8, 25]. We expect that students who are motivated by an intellectual quest and aspire an academic career will be more focused on contributing to the academic community and outperforming their peers. For them, not only is the PhD itself important, but also getting published, visiting conferences, and other activities that will create valuable academic resume. We expect these PhD students to be affected most and thus to expect more support and to assess this support stricter, which will result in a decrease of satisfaction scores over time (**H4**).

#### Living situation

The different living situations of PhD students are impacted differently by the COVID-19 restrictions. PhD students that live alone face the consequences of social isolation due to restrictions that limit social contact, whereas PhD students living with children face the challenging consequence of combining working from home with family life that, mainly due to school closures, was completely withdrawn into the domestic sphere too. Expectations for supervisor support may change depending on the extent to which the family context is affected by the COVID-19 restrictions. In line with earlier findings [8], we therefore expect the score of satisfaction with supervisor support not only to vary between PhD students in different living situations (**H5a**), but also that the change in score over time is stronger for PhD students that live with children or in other living situations compared to PhD students that live with a partner only (**H5b**).

#### Nationality

The social restrictions due to the COVID-19 pandemic, such as shutting down campus life, the very restrictive conditions under which it is possible to meet with friends or family and the ban on non-essential travel abroad, have an impact on the social supportive network of PhD students. We consider foreign PhD students to be extra vulnerable for these consequences that, in turn, might have repercussions on doing doctoral research and result in the need for (extra) support and understanding from supervisors. In line with earlier expressed concerns [3], we therefore expect foreign PhD students’ assessment of supervisor support to be stricter and thus lead to a decrease in satisfaction scores over time (**H6**).

#### Gender

Pre-COVID-19, more female than male PhD students reported higher levels of anxiety and depression [30] and more stress [33]. During the COVID-19 pandemic women, and especially women with caregiving responsibilities, start to publish less [28] and, out of necessity, have to prioritize their time in ways that are unfavorable for their future careers [27]. Although research did not report a difference in the share of women that reported worsened supervisor support compared to those whose support remained unchanged [7], we do expect women’s assessment of supervisor support to be stricter and thus lead to a stronger decrease in satisfaction scores over time when compared to their male counterparts (**H7**).

## Data & method

This study relies on data from the PhD Survey of the Vrije Universiteit Brussel (VUB). The VUB is located in the Brussels Capital Region, which is both part of the French and Flemish Community of Belgium. The registration of these language communities in the Belgian Constitution in 1970 implied the establishment of so-called cultural communities that are given the power to regulate language use regarding, for example, education. As a result, the VUB has been legally and officially recognised as the Dutch-speaking university in Brussels alongside the French-speaking university since 1970, but both universities have their joint origin in the French speaking Université Libre de Bruxelles that was founded in 1834.

In the academic year 2019–2020 just over 19,000 students were enrolled in 149 study programmes of which one third is taught in English. About 10% of enrolled students are enrolled in PhD programmes. The general admission requirements to conduct doctoral research at the VUB (and any other Flemish university) include possession of a recognized master’s degree, the need of a supervisor, and the need of funding. PhD students in Belgium can rely on different funding opportunities, such as general or themed scholarships from (inter)national funding institutions (e.g., the national research council), research funding from a research project or multiple research projects in the name of the supervisor, or by combining PhD research with a position as teaching assistant.

PhD students enrol in the compulsory Doctoral Training Programme which facilitates PhD students with the possibility to develop their (research) skills through, for example, courses, seminars, workshops, and career coaching. There are three different doctoral schools under which all faculties are divided. The *Doctoral School of Natural Sciences and (Bioscience) Engineering* (NSE) includes the Faculty of Engineering Sciences and the Faculty of Sciences & Biosciences engineering. The *Doctoral School of Human Sciences* (DSh) includes the Faculty of Social Sciences & Solvay Business School, the Faculty of Arts & Philosophy, the Faculty of Psychology & Educational Science, and the Faculty of Law & Criminology. The *Doctoral School of Life Science and Medicine* (LSM) includes the Faculty of Medical Sciences & Pharmacy and the Faculty of Physical education & Physiotherapy.

Doctoral research typically lasts for four years and ends with a successful oral defense of the thesis.

### PhD survey

In the empirical part of the study, we rely on data from the PhD Survey. This annual survey is commissioned by the Research, Training & Development Office (RTDO) at the VUB and conducted by the Research Group TOR (Tempus Omnia Revelat) at the same university. The PhD Survey serves as a monitor-instrument to evaluate the support provided to PhD students by RTDO and at the same time monitor aspects of well-being and job satisfaction of PhD students.

A pilot of the PhD Survey among a limited number of faculties took place in the springtime of 2017 (wave 0). Since 2018 onwards, the PhD Survey is being conducted university wide and the 4^th^ wave has been completed in 2021. The PhD Survey is longitudinal in its design since it aims to follow PhD students throughout their PhD trajectory, which typically lasts four years. Attrition can be attributed to PhD students quitting or successfully finishing their PhD, or non-response to one or more waves. For privacy reasons we have no access to administrative data that would enable to distinguish between these different types of attrition. Influx is natural and based on the number of new PhD students registered at the VUB on the 1^st^ of January preceding the launch of the next wave. Typically, PhD Students start in October or November, but it is possible to start at any time of the academic year.

The PhD Survey exists of a single online questionnaire that is hosted on the data collection platform MOTUS and accessible through the MOTUS web application [34]. The PhD Survey generally takes place in the last two weeks of April and the whole month of May. PhD students across all faculties receive an email with login credentials to participate in the survey. Up to two reminders are sent, eight and 20 days after the day of initial invitation. Additionally, the PhD Survey is advertised in the monthly PhD newsletter in the months preceding the PhD Survey and, between reminders, group emails are sent at the faculty level.

Based on the rules of the own institution at the time of PhD Survey waves 0 (2017) to 4 (2021), no advice from the ethics committee is required for an internal survey. Nevertheless, the PhD Survey follows common ethical aspects. PhD students were informed in the emails about the aim of the study, about how data will be used and how feedback can be obtained, and who to contact for further questions and technical support. The emails included information and links about the study’s privacy statement and the general privacy statement of the software platform used to administer the survey. PhD students consented to the survey by clicking on the link in the emails and using their username and password to login to the software platform.

This contribution uses data from wave 2 held in 2019 [35], wave 3 held in 2020 [36] and wave 4 held in 2021 [37]. Response rates are 44.9%, 44.3% and 42.8%, respectively, which is in line with other surveys on PhD students [25]. We created two cohorts. Cohort 1 exists of all PhD students that responded to both the 2019 and 2020 editions of the PhD Survey (n = 345). This cohort represents a pre-COVID measurement (April-May 2019) and a measurement (April-May 2020) that followed immediately after the start of the abrupt lockdown that lasted from March till May 2020. Cohort 2 comprises all PhD students that responded to both the 2020 and 2021 editions of the PhD Survey (n = 349). This cohort represents a measurement at the onset of the pandemic (April-May 2020) and a measurement (April-May 2021) after a year with continuously changing containment policies. The construction of two cohorts is motivated by the hypothesized difference of impact from the COVID-19 pandemic and sample size maximisation. The first cohort represents PhD students that were abruptly impacted for an intense and short period. The second cohort represents PhD students whose research was impacted by a year of alternating tightening and easing of restrictions.

Although feasible, a three-wave study would only contain 167 PhD students. Moreover, due to privacy regulations, no administrative data on completion of or drop-out from the PhD trajectory is available. This makes it hard to evaluate attrition. Indeed, it cannot be known whether a PhD student is a first-year graduate in 2020 or simply did not respond to the survey of 2019.

### Variables

The dependent variable is PhD students’ *satisfaction with supervisor support*. Satisfaction with supervisor support is measured by six items that are rated on a 5-point Likert scale ranging from *not at all satisfied* (1) to *very satisfied* (5). The items inquire satisfaction with supervisor’s involvement in the research, expertise, support, and stimulation to solve research related issues, as well as the frequency and the quality of meetings. For all waves, principal component analyses revealed one component with eigenvalues greater than one (see Table 1). The interpretation of the data was consistent with satisfaction with supervisor support the items were designed to measure. For all waves, the scale had a high level of internal consistency, as determined by Cronbach’s alpha (0.88 < α‘s < 0.89). Table 1 shows the component loadings, eigenvalues, and Cronbach’s alphas for both cohorts. To assure equal contribution of each item to the composite measure of satisfaction with supervisor support, we construct a summation scale *T* that ranges from 0 to 10 using the following equation:

$$T=\frac{\left(s_{1}+s_{2}+\cdots +s_{i})-min(s_{1}+s_{2}+\cdots +s_{i}\right)}{(S-1)\times N_{i}}\times 10$$ (1)

where the total score *T* is the result of multiplying 10 by the summation of the respondent’s answers *s* on all items *i* minus the minimum summation of answers *s* on all items *i*, divided by the total number of answering possibilities per item *S* minus 1 times the total number of items *N*_*i*_. This strategy was preferred over a structural equation model analysis with equality constraints on the factor loadings for the different waves, because in the main analysis we are interested in moderation effects which can be presented in a more intuitive way with regression analysis. Moreover, the summation scales correlated very highly with the factors obtained from PCA analysis (r’s > 0.99).

**Table 1:** Eigenvalue and Cronbach’s alpha for component of satisfaction with supervisor support by year of PhD survey.

Items^†^	Cohort 1		Cohort 2	
To what extent are you satisfied with:	2019	2020	2020	2021
Stimulation/inspiration to solve research problems/issues	0.774	0.830	0.849	0.857
The quality of meetings	0.836	0.847	0.847	0.813
The expertise she/he has on the research subject	0.793	0.799	0.793	0.777
The support you receive in writing articles	0.748	0.758	0.727	0.809
The frequency of meetings	0.783	0.781	0.774	0.780
Involvement of your supervisor(s) in your research?	0.762	0.753	0.736	0.702
Eigenvalue	4.84	4.87	4.84	4.88
Cronbach’s alpha	0.88	0.89	0.88	0.88

^†^Answering options: not at all satisfied, rather not satisfied, undecided, rather satisfied, very satisfied.

The main independent variable of interest is *time*. To assess the net effect of *time*, the statistical models control for socio-demographic characteristics of the PhD students, as well as objective and subjective characteristics of PhD students’ work environment. Socio-demographic characteristics includes a dummy for *female*, a dummy for *Belgian nationality*, and *living situation* (with partner [reference category], with children, other). Note that the category ‘with children’ includes both PhD students who are a single parent and PhD students that form a two-parent family. The category ‘other’ includes PhD students that live alone, with their parents, or in student houses or other shared housing.

Characteristics of work environment include membership of *doctoral school* (Doctoral School of Natural Sciences and (Bioscience) Engineering [NSE, reference category], Doctoral School of Human Sciences [DSh], Doctoral School of Life Science and Medicine [LSM]) to measure discipline, a dummy for whether the *PhD is in the finalizing phase* (self-defined), and a dummy for *expected to work in academia*. The latter variable is used as a proxy for the more general frame of reference and motivation of PhD students [31]. PhD students who aim to stay in academia know that they not only have to write an excellent PhD thesis, but also (intellectually) contribute to the academic community by trying to publish several journal articles, present at important conferences, and outperform their peers.

Table 2 provides an overview of the distribution of the socio-demographic and job characteristics for both cohorts.

**Table 2:** Distribution of the socio-demographic characteristics and job characteristics by cohort.

Characteristics		Cohort 1	Cohort 2
		(n = 345)	(n = 349)
**Socio-demographic characteristics**			
Sex (%)	*Male*	47.7	45.8
	*Female*	53.3	54.2
Nationality (%)	*Belgian*	47.8	46.2
	*Non-Belgian*	52.2	53.8
Living situation (%)	*With partner*	44.9	46.2
	*With children*	18.6	14.8
	*Other*	36.5	39.0
**Job characteristics**			
Doctoral school (%)^†^	*NSE*	42.6	40.2
	*DSh*	35.3	33.0
	*LSM*	22.2	26.7
Phase of PhD (%)	*Not in finalizing phase*	67.5	70.1
	*Finalizing phase*	32.5	29.9
Expect to work in academia (%)	*No*	67.8	61.6
	*Yes*	32.2	38.4

^†^Doctoral School of Natural Sciences and (Bioscience) Engineering (NSE), Doctoral School of Human Sciences (DSh), Doctoral School of Life Science and Medicine (LSM).

### Analysis plan

The analysis proceeds in two steps. First, we provide descriptive statistics of (the changes in) the item scores that measure satisfaction with supervisory support as well as (changes in) the means of the composite measure. The Likert-item scores are considered an ordinal approximation of a continuous variable and therefore presented as means with a minimum of 1 and maximum of 5 [see discussions in 38, 39]. The descriptive analyses are presented for both cohorts separately and tested for statistically significant differences between groups within cohorts using paired-sample t-tests. Given the relatively small sample size, the threshold for statistical significance is set at α = 0.10.

Second, we use one-way repeated measures ANOVA to assess the association between *time* and *satisfaction with supervisory support* net of socio-demographic characteristics and job characteristics. Statistical models are presented for both cohorts separately. In these models we first test for between-subject effects. Subsequently, we study the within-subject time effect. Then, we test for within-subject time interaction effects. In the final model we present all relevant between-subject and within-subject effects.

## Results

### Descriptive results

Table 3 shows the mean scores and standard deviations for the items underlying the composite measure and the score on the composite measure for satisfaction with supervisor support. For cohort 1, the mean scores for stimulation/inspiration to solve research problems/issues by the supervisor, the expertise the supervisor has on the research subject, and the extent to which the supervisor is involved in the research were significantly lower in 2020 during the first lockdown compared to 2019. The other items, albeit not significant, showed a similar tendency towards a decreased satisfaction. As a result, the mean score of the composite measure for satisfaction with supervisor support dropped significantly between 2019 and 2020 from 7.360 in 2019 to 7.171 in 2020. This provisionally confirms **H1a**.

**Table 3:** Descriptive results scores on items on satisfaction with supervisor support and composite measure of overall satisfaction with supervisor support by cohort and years within cohort.

**Cohort 1**	**2019**	**2020**	**|Diff.|**	**Sig.**
*Items* ^*†*^	*mean (standard deviation)*			
Stimulation/inspiration to solve research problems/issues	3.8 (1.0)	3.7 (1.1)	0.10 (1.07)	(*)
The quality of meetings	3.9 (1.0)	3.8 (1.1)	0.04 (0.94)	^n.s.^
The expertise she/he has on the research subject	4.1 (1.0)	4.0 (1.0)	0.12 (0.84)	**
The support you receive in writing articles	3.9 (1.1)	3.9 (1.1)	0.03 (1.03)	^n.s.^
The frequency of meetings	3.9 (1.0)	3.8 (1.1)	0.06 (0.95)	^n.s.^
Is/are your supervisor(s) involved in your research?	4.1 (1.0)	4.0 (1.0)	0.10 (0.86)	*
*Composite measure* ^*‡*^				
Satisfaction with supervisor support	7.4 (2.0)	7.2 (2.1)	0.19 (1.66)	*
**Cohort 2**	**2020**	**2021**	**|Diff.|**	**Sig.**
*Items* ^*†*^	*mean (standard deviation)*			
Stimulation/inspiration to solve research problems/issues	4.0 (1.0)	3.9 (1.0)	0.07 (0.91)	^n.s.^
The quality of meetings	4.1 (1.0)	4.1 (1.0)	0.02 (0.97)	^n.s.^
The expertise she/he has on the research subject	4.3 (0.9)	4.2 (0.9)	0.05 (0.90)	^n.s.^
The support you receive in writing articles	4.0 (1.0)	4.0 (1.1)	0.02 (0.98)	^n.s.^
The frequency of meetings	4.0 (1.0)	3.9 (1.1)	0.07 (0.98)	^n.s.^
Is/are your supervisor(s) involved in your research?	4.3 (0.9)	4.2 (1.0)	0.08 (0.85)	(*)
*Composite measure* ^*‡*^				
Satisfaction with supervisor support	7.8 (1.9)	7.6 (2.0)	0.12 (1. 60)	^n.s.^

Note. |Diff.| = absolute mean difference between years. Sig. = two-sided significance of difference between years based on paired-sample t-test. Levels of significance

***p≤0.001

**p≤0.01

*p≤0.05

(*)p≤0.10, n.s. not significant.

^†^Items can take values from 1 to 5. Higher values indicate greater degree of satisfaction.

^‡^Composite measure can take values from 1 to 10. A higher value indicates a greater degree of overall satisfaction with supervisor support.

For cohort 2, only the mean scores for the satisfaction with the involvement of the supervisor in PhD research was significantly lower in 2021 compared to 2020. Again, almost all other items, albeit not significant, showed a similar tendency towards decreased satisfaction. The mean score of the composite measure for satisfaction with supervisor support dropped between 2020 and 2021 from 7.752 to 7.634, However, this difference was not statistically significant. This provisionally confirms **H1b**. We note that the average satisfaction score for 2020 in the first cohort was substantially lower than the average satisfaction score for 2020 in the second cohort. This might be ascribed to attrition caused by a healthy worker effect [40].

### Multivariate results

Table 4 shows the results of the changes in the composite measure of satisfaction with supervisor support for cohort 1 (2019 *vs*. 2020). The partial *η* is an indication of the strength of an association and reads like a standardised regression coefficient [41]. Higher values reflect stronger associations. The Cohen’s *d* is an indicator of the effect size and expresses how many standard deviations lie between two means. Higher values imply larger effect sizes.

**Table 4:** Results of one-way repeated measure ANOVA for composite measure of satisfaction with supervisor support for cohort 1.

	Step 1^†^			Step 2^†^			Step 3^†^			Step 4^‡^		
	Cohen’s *d*	Partial *η*	Sig.	Cohen’s *d*	Partial *η*	Sig.	Cohen’s *d*	Partial *η*	Sig.	Cohen’s *d*	Partial *η*	Sig.
**Between-subjects effects**												
Living situation (*ref*. *alone*)		0.122	(*)	/	/	/	/	/	/		0.088	^n.s.^
With children	-0.311									-0.115		
With partner only	-0.228									-0.127		
PhD in finalizing phase (*ref*. *no*)		0.108	(*)	/	/	/	/	/	/		0.079	^n.s.^
Yes	0.233									0.135		
Expect academic career (*ref*. *no*)		0.018	^n.s.^	/	/	/	/	/	/		0.197	***
Yes	-0.038									0.340		
**Within-subjects main effect**												
Time	/	/	/	-0.114	0.114	*	/	/	/	-0.077	0.078	^n.s.^
**Within-subjects interaction effects**												
Time*Living situation (*ref*. *alone*)	/	/	/	/	/	/		0.122	(*)		0.145	*
Time*With children							-0.285			-0.208		
Time*With partner only							-0.187			-0.107		
Time*Alone							0.039			0.090		
Time*PhD in finalizing phase (*ref*. *no*)	/	/	/	/	/	/		0.108	(*)		0.121	*
Time*Yes							0.040			0.030		
Time*No							-0.185			-0.154		
Time*Expects academic career (*ref*. *no*)	/	/	/	/	/	/		0.018	^n.s.^		0.032	^n.s.^
Time*Yes							-0.010			-0.088		
Time*No							-0.008			-0.045		

Levels of significance

***p≤0.001

**p≤0.01

*p≤0.05

(*)p≤0.10, n.s. not significant

^†^Separate models; / = not included in this step

^‡^Full model.

Step 1 looks at the between-subject effects in the difference of satisfaction with supervisor support. This difference varied significantly by living situation. The Cohen’s *d* indicates that the satisfaction with supervisor support decreased substantially for PhD students living with children and for PhD students living with a partner compared to PhD students with other living situations. PhD students that are in the finalizing phase of their PhD research were more satisfied with supervisor support than their peers that are still in the starting or executing phase of their PhD research. Step 2 reports a decrease in supervisor support over time. Step 3 shows that this time-effect was larger for PhD students living with children or with partner and for PhD students that are not in the finalizing phase of their PhD research. The absence of any associations by doctoral schools, sex, and nationality confirms **H2** and rejects **H6** and **H7** for cohort 1. There were no differences in the decrease of score of satisfaction with supervisor support between the doctoral schools, between Belgian and non-Belgian PhD students, and between men and women.

Step 4 presents the final multivariate model. The initial between-subject effects of living situation and being in the finalizing phase of PhD research and main effect for time were no longer significant. This leads us to reject **H1a** and **H3a** and **H5a**. Instead, satisfaction with supervisor scores differed within categories of living situations and phase of PhD research over time. **H5b** is partially confirmed. PhD students living with children were significantly less satisfied with support from their supervisor. However, it was not the PhD students in other living situations but the PhD students living with a partner that were significantly less satisfied with support from their supervisor. **H3b** is also confirmed. PhD students that are not in the finalizing phase of their PhD research were significantly less satisfied with support from their supervisor. Keeping constant variations over time within living situation and phase of PhD research led to a highly significant and substantial effect of the expectancy to work in academia. PhD students that expect to work in academia reported a smaller decrease in their satisfaction with supervisor support compared to their peers that do not expect to work in academia or are undecided. This not only rejects **H4,** but also inverts it.

We applied the same analytical strategy to cohort 2 for the comparison between 2020 and 2021. Only the expectancy to work in academia yielded significant effects (results not shown), which, again, is an inversion of **H4**. Like cohort 1, PhD students that do not expect to work in academia reported a significantly lower score of satisfaction with supervisor support (*η* = 0.288, *p*<0.001). Unlike cohort 1, there was also an interaction effect with time (*η* = 0.098, *p* = 0.073) indicating that the decrease in the score of satisfaction with supervisor support was significantly larger for PhD students that do not expect to work in academia compared to their peers who pursue an academic career. All the other hypotheses are rejected.

**Fig 1:**
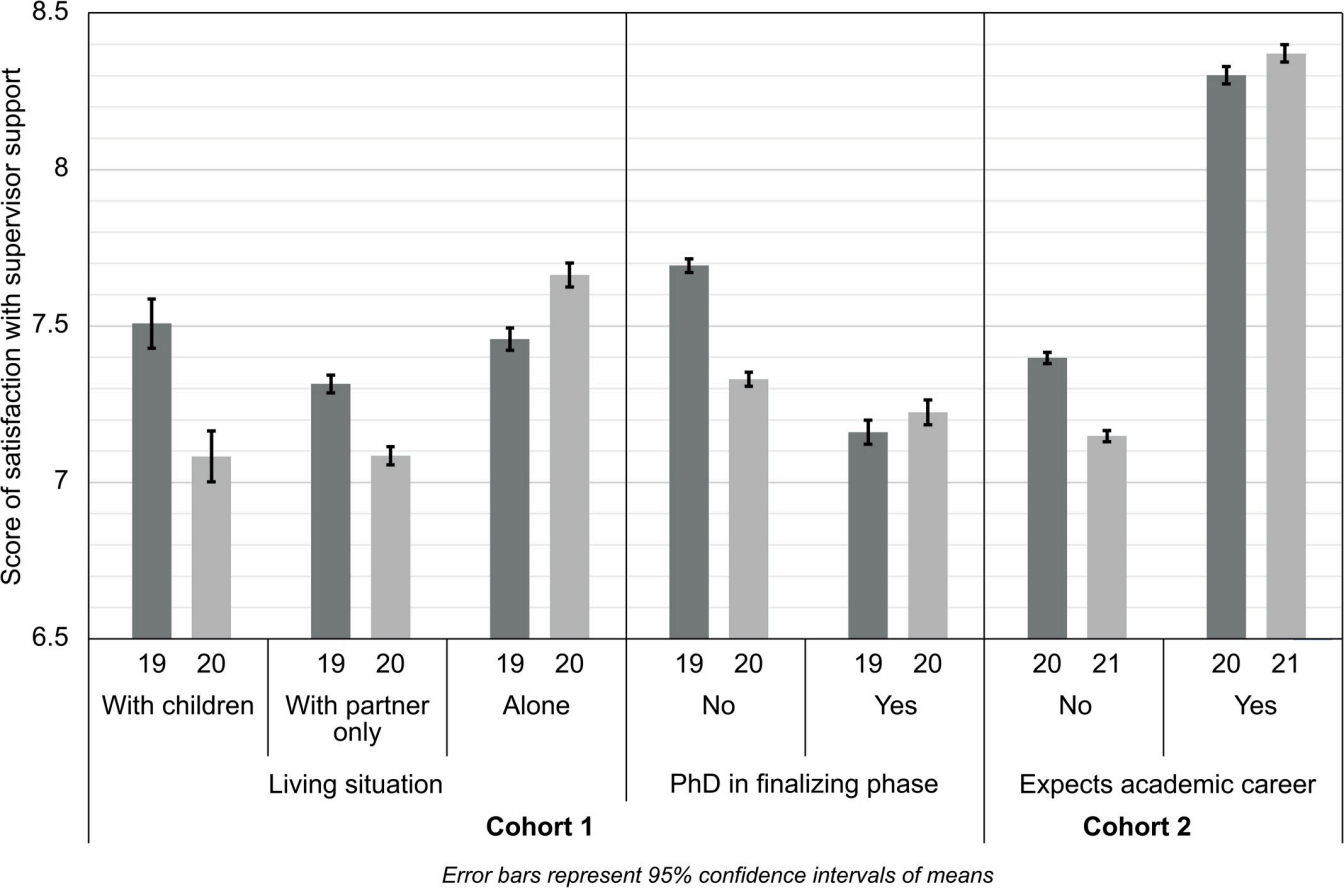
Estimated marginal means of score of satisfaction with supervisor support over time.

Fig 1 summarizes the interaction terms with time. For cohort 1, it clearly shows the substantial decrease in the satisfaction score *within* PhD students living with children, PhD students living with a partner only, and PhD students that are not in the finalizing phase of their PhD research pre-COVID in 2019 and during the lockdown of 2020. For cohort 2, it not only shows the substantial difference *between* PhD students that expect and do not expect an academic career on this score, but also the substantial decrease on the score of satisfaction with supervisor support over time *within* PhD students that do not expect an academic career.

## Discussion

PhD students’ satisfaction with supervisor support is an important indicator of their well-being. It reflects how well they feel supported in doing their doctoral research. This support came under pressure during the COVID-19 pandemic [1–4]. PhD students’ already high stress levels [28] might increase even further by the consequences of the COVID-19 pandemic [42]. Many PhD students found themselves in situations that might have given rise to increased need of supervisor support. In line with existing research on the challenges of the COVID-19 pandemic for PhD students [7, 8, 23–28], we found a significant decrease in PhD students’ satisfaction scores with supervisor support over time between 2019 and the onset of the COVID-19 pandemic in 2020 (i.e., cohort 1). However, multivariate analyses showed that this drop was caused by different groups of PhD students, which concurs with research that categorizes PhD students’ worries and concerns in three categories: personal concerns, disruption of research activities, and career impact [8]. Firstly, personal concerns, measured in this study as a challenging living situation, is most strongly associated with decreasing satisfaction with supervision This is in line with findings that report PhD students’ concern about health of friends and family [8] and findings about the daily family struggles of the COVID-19 restrictions [43, 44]. Indeed, PhD students that live with children reported the largest drop in satisfaction scores. Similarly, PhD students that live with a partner only, also reported a substantial drop in satisfaction scores. This suggests that it is the inflexibility and unpredictability that stems from being responsible for or taking into consideration other family members during a lockdown that causes a mismatch between expected and provided support by supervisors.

Secondly, PhD students that just got started or were gathering data reported a substantial drop in satisfaction scores over time. Satisfaction scores of PhD students in their finalizing phase of their doctoral research remained unchanged. Junior PhD students might have a higher need for support to become acquainted with the research group and meeting colleagues, to kick-off a research agenda, or to change a research plan vis-à-vis data collection method and period. In other words, PhD students’ ignorance and uncertainty seem to play an important role in their assessment of–and consequent decrease in–the satisfaction score. This result suggests that the disruption of research activities due to the COVID-19 pandemic impacted junior PhD students most. Other studied consequences, such as the unproductivity of mandatory telework [4] and risk of working overtime [8, 23] might be equally stratified by PhD students’ seniority.

Thirdly, PhD students that ambition an academic career were not less satisfied with supervisor support measured over a period of COVID-related measures between 2020 and 2021 (i.e., cohort 2). This was unexpected because other studies revealed PhD students’ increased concerns about career impact [8, 25], which might give rise to a higher need for support. More worrisome is that PhD students without the ambition to pursue an academic career were much less satisfied with supervisor support over the same period. In other words, it seems that in a year of varying severity of COVID-19 restrictions and its impact on doing doctoral research, PhD students without an ambitious academic frame of reference are experiencing the negative impact of the COVID-19 pandemic to a greater extent.

The latter finding aligns with the idea that PhD students’ motivation differ and that the resulting expectations cannot be met with a single approach [31]. Indeed, this study also suggests that PhD students approach their doctoral research from at least two different frames of reference: as a trajectory of formation, learning and self-development versus an unconditional step to position themselves in the academic world. Both approaches require different levels of support from supervisors (and by extension from universities) and it is not inconceivable that the latter type of PhD student is easier to support in crisis situations than the former.

These findings point to policy challenges vis-à-vis PhD students. The results of the analyses of the first cohort clearly show that the COVID-19 pandemic has the potential to reinforce inequality and that a differentiated policy is needed to create and maintain a level playing field. Scholars indeed report on the need for both generalised and specific support running from financial assistance to mental health and pastoral support [45] and the need to follow up on existing support and/or identifying new forms of support for PhD students would be beneficial [46]. However, the results of the second cohort reveal much less inequality. This raises the question how stable the results of the first cohort are and whether the impact of the COVID-19 pandemic varies across different phases of the pandemic (and within subgroups). This stresses the importance of cross-sectional or longitudinal follow up on this matter. In this study, the next wave may shed more light on this, but if the impact of the COVID-19 pandemic does indeed vary as the pandemic continues and is contained, it makes it more difficult to implement policies to mitigate the impact hereof.

This contribution is not without its limitations. The COVID-19 restrictions not only directly and indirectly impacted PhD students, but also supervisors themselves. The results clearly point in the direction that for some groups of PhD students, supervisor support during the COVID-19 pandemic was insufficient. It is conceivable that the mismatch between support not only arose from changing expectations from PhD students, but also from work-related challenges, such as online teaching, recording lessons, organizing exams in a safe way, and family- or health-related challenges amongst supervisors. Although supervisors have an important responsibility towards their PhD students, we do not want to underestimate the impact of the COVID-19 pandemic on themselves at any point. Future editions of the PhD Survey would benefit from further contextualization by at least investing the expectations of PhD students *and* those of their supervisors. Additionally, in its current form, not much is known about attrition of the sample. PhD students that faced a severe impact from the COVID-19 pandemic on their (work-)life and judged the support from their supervisor, and by extension the university, insufficient, might have dropped out between the 2020 and 2021 PhD Survey data collection. Linking future editions with university’s administrative data would provide more information about attrition due to drop-out *versus* successful completion *versus* non-response in earlier waves.

## Conclusion

PhD students’ satisfaction with supervisor support is an important indicator of their well-being. This study did not show a main effect of time on satisfaction with supervisor support following the unprecedent restrictions at the onset of the COVID-19 pandemic between 2019 and 2020 (reject H1a) nor after a year of relaxed and tightened restrictions between 2020 and 2021 (reject H1b). However, substantial interaction effects of time showed a stronger negative impact on satisfaction with supervisor support of the COVID-19 pandemic over time for PhD students who start their doctoral research or conduct or plan data collection (accept H3b) and PhD students in living situations in which they bear multiple responsibilities (accept H5b) between 2019 and 2020, and for PhD students who do not expect a career in academia (accept H4) between 2020 and 2021. No interactions of time were found for doctoral schools (accept H2) indicating that PhD students in all university departments faced COVID-19 related challenges, or for nationality (reject H6) and gender (reject H7). Finally, satisfaction with supervisor score did not vary between PhD students in different phases of their PhD trajectory (reject H3a) or in different living situations (reject H5a) regardless time.

In times of crises, which affects both PhD students and supervisors, special attention needs to be paid to PhD students who are extra susceptible to uncertainties because of their junior status or personal situation, and especially those PhD students for whom doctoral research is a trajectory of formation and self-development instead of a steppingstone to position themselves in academia.

## References

[1] Aydemir D, Ulusu NN. Commentary: Challenges for PhD students during COVID‐19 pandemic: Turning crisis into an opportunity. Biochem Mol Biol Educ. 2020;48(5):428–9. doi: 10.1002/bmb.21351 32271978 PMC7262270

[2] Chan C, Oey NE, Tan E-K. Mental health of scientists in the time of COVID-19. Brain Behav Immun. 2020;88:956. doi: 10.1016/j.bbi.2020.05.039 32405151 PMC7219424

[3] Cheng C, Song S. How early-career researchers are navigating the Covid-19 pandemic. Mol Plant. 2020;13(9):1229. doi: 10.1016/j.molp.2020.07.018 32730995 PMC7384411

[4] Paula JR. Lockdowns due to COVID-19 threaten PhD students’ and early-career researchers’ careers. Nat Ecol Evol. 2020;4(8):999. doi: 10.1038/s41559-020-1231-5 32493949 PMC7392882

[5] Elias S. Cultivating mentorship, cooperation, and community during COVID-19 and beyond. Cell Stem Cell. 2021;28(5):802–4. doi: 10.1016/j.stem.2021.04.016 33961764 PMC9071456

[6] Lee A. How are doctoral students supervised? Concepts of doctoral research supervision. Stud High Educ. 2008;33(3):267–81.

[7] Börgeson E, Sotak M, Kraft J, Bagunu G, Biörserud C, Lange S. Challenges in PhD education due to COVID-19-disrupted supervision or business as usual: a cross-sectional survey of Swedish biomedical sciences graduate students. BMC Med Educ. 2021;21(1):1–11. 34022871 10.1186/s12909-021-02727-3 PMC8140581

[8] Suart C, Nowlan Suart T, Graham K, Truant R. When the labs closed: graduate students’ and postdoctoral fellows’ experiences of disrupted research during the COVID-19 pandemic. FACETS. 2021;6(1):966–97.

[9] Dericks G, Thompson E, Roberts M, Phua F. Determinants of PhD student satisfaction: the roles of supervisor, department, and peer qualities. Assess Eval High Educ. 2019;44(7):1053–68.

[10] Evans TM, Bira L, Gastelum JB, Weiss LT, Vanderford NL. Evidence for a mental health crisis in graduate education. Nat Biotechnol. 2018;36(3):282–4. doi: 10.1038/nbt.4089 29509732

[11] McCallin A, Nayar S. Postgraduate research supervision: A critical review of current practice. Teach High Educ. 2012;17(1):63–74.

[12] Heath T. A quantitative analysis of PhD students’ views of supervision. High Educ Res Dev. 2002;21(1):41–53.

[13] Åkerlind G, McAlpine L. Supervising doctoral students: Variation in purpose and pedagogy. Stud High Educ. 2017;42(9):1686–98.

[14] Bedggood RE, Donovan JD. University performance evaluations: what are we really measuring? Stud High Educ. 2012;37(7):825–42.

[15] Zhao CM, Golde CM, McCormick AC. More than a signature: How advisor choice and advisor behaviour affect doctoral student satisfaction. J Furth High Educ. 2007;31(3):263–81.

[16] Taylor RT, Vitale T, Tapoler C, Whaley K. Desirable qualities of modern doctorate advisors in the USA: a view through the lenses of candidates, graduates, and academic advisors. Stud High Educ. 2018;43(5):854–66.

[17] Gunnarsson R, Jonasson G, Billhult A. The experience of disagreement between students and supervisors in PhD education: a qualitative study. BMC Med Educ. 2013;13(1):1–8. doi: 10.1186/1472-6920-13-134 24074051 PMC3851003

[18] Hockey J. Establishing boundaries: problems and solutions in managing the PhD supervisor’s role. Camb J Educ. 1994;24(2):293–305.

[19] Zúniga-Gonzalez CA. The role of the mediator and the student in the face of new educational scenarios: COVD-19. Revista Electrónica Calidad En La Educación Superior. 2021;12(2):279–94.

[20] Heidari E, Mehrvarz M, Marzooghi R, Stoyanov S. The role of digital informal learning in the relationship between students’ digital competence and academic engagement during the COVID‐19 pandemic. J Comput Assist Learn. 2021;37(4):1154–66. doi: 10.1111/jcal.12553 34230741 PMC8250506

[21] Omar HA, Ali EM, Belbase S. Graduate Students’ Experience and Academic Achievements with Online Learning during COVID-19 Pandemic. Sustainability. 2021;13(23):13055.

[22] van Rooij E, Fokkens-Bruinsma M, Jansen E. Factors that influence PhD candidates’ success: the importance of PhD project characteristics. Stud Contin Educ. 2021;43(1):48–67.

[23] Donohue WJ, Lee AS-J, Simpson S, Vacek K. Impacts of the COVID-19 pandemic on doctoral students’ thesis/dissertation progress. Int J Dr Stud. 2021;16:533–52.

[24] Glorieux A, van Tienoven TP, te Braak P, Minnen J, Spruyt B. Doing PhD research during the COVID-19 pandemic. First results of the PhD Survey 2021. Vrije Universiteit Brussel, 2021 Available from: https://torvub.be/torwebdat/publications/t2021_28.pdf.

[25] Haas N, Gureghian A, Díaz CJ, Williams A. Through their own eyes: The implications of COVID-19 for PhD students. J Exp Political Sci. 2020:1–21.

[26] Johnson RL, Coleman RA, Batten NH, Hallsworth D, Spencer EE. The Quiet Crisis of PhDs and COVID-19: Reaching the financial tipping point. Research Square. 2020. doi: 10.21203/rs.3.rs-36330/v2

[27] Minello A, Martucci S, Manzo LK. The pandemic and the academic mothers: present hardships and future perspectives. Eur Soc. 2021;23(sup1):S82–S94.

[28] Viglione G. Are women publishing less during the pandemic? Here’s what the data say. Nature. 2020;581(7809):365–7. doi: 10.1038/d41586-020-01294-9 32433639

[29] Pilbeam C, Lloyd-Jones G, Denyer D. Leveraging value in doctoral student networks through social capital. Stud High Educ. 2013;38(10):1472–89.

[30] Stecker T. Well‐being in an academic environment. Med Educ. 2004;38(5):465–78. doi: 10.1046/j.1365-2929.2004.01812.x 15107080

[31] Reasons Skakni I., motives and motivations for completing a PhD: a typology of doctoral studies as a quest. Stud Grad Postdr Educ. 2018;9(2):197–212.

[32] Naylor R, Chakravarti S, Baik C. Differing motivations and requirements in PhD student cohorts: A case study. Issues Educ Res. 2016;26(2):351–67.

[33] Ulku-Steiner B, Kurtz-Costes B, Kinlaw C. Doctoral student experiences in male-dominated and gender-balanced graduate programs. J Educ Psychol. 2000;92:296–307.

[34] MOTUS. MOTUS research platform 2016–2021. Available from: https://www.motusresearch.io/en.

[35] Glorieux A, te Braak P, Minnen J, Spruyt B. PhD Survey VUB 2019: Technical Report. Vrije Universiteit Brussel, 2019 Available from: https://torvub.be/torwebdat/publications/t2020_11.pdf.

[36] Glorieux A, te Braak P, Laurijssen I, Van Deynze F, De Grande H, Minnen J, et al. PhD Survey VUB 2020: Technical Report. Vrije Universiteit Brussel, 2020 Available from: https://torvub.be/torwebdat/publications/t2021_15.pdf.

[37] Glorieux A, van Tienoven TP, Spruyt B, te Braak P, Minnen J. PhD Survey VUB 2021: Technical Report. Vrije Universiteit Brussel, 2021 Available from: https://torvub.be/torwebdat/publications/t2022_10.pdf.

[38] Norman G. Likert scales, levels of measurement and the “laws” of statistics. Adv Health Sci Educ Theory Pract. 2010;15(5):625–32. doi: 10.1007/s10459-010-9222-y 20146096

[39] Sullivan GM, Artino AR Jr. Analyzing and interpreting data from Likert-type scales. J Grad Med Educ. 2013;5(4):541–2. doi: 10.4300/JGME-5-4-18 24454995 PMC3886444

[40] Shah D. Healthy worker effect phenomenon. Indian J Occup Environ Med. 2009;13(2):77. doi: 10.4103/0019-5278.55123 20386623 PMC2847330

[41] Ellis PD. The essential guide to effect sizes: Statistical power, meta-analysis, and the interpretation of research results: Cambridge University Press; 2010.

[42] Paucsik M, Leys C, Marais G, Baeyens C, Shankland R. Self‐compassion and savoring buffer the impact of the first year of the COVID‐19 on PhD students’ mental health. Stress Health. 2022. doi: 10.1002/smi.3142 35286765 PMC9111133

[43] Craig L, Churchill B. Working and caring at home: Gender differences in the effects of COVID-19 on paid and unpaid labor in Australia. Fem Econ. 2021;27(1–2):310–26.

[44] Manzo LKC, Minello A. Mothers, childcare duties, and remote working under COVID-19 lockdown in Italy: Cultivating communities of care. Dialogues Hum Geogr. 2020;10(2):120–3.

[45] Goldstone R, Zhang J. Postgraduate research students’ experiences of the COVID-19 pandemic and student-led policy solutions. Educ Rev (Birm). 2021. doi: 10.1080/00131911.2021.1974348

[46] Varadarajan J, Brown AM, Chalkley R. Biomedical graduate student experiences during the COVID-19 university closure. PLoS One. 2021;16(9):e0256687. doi: 10.1371/journal.pone.0256687 34529681 PMC8445460

